# Poly[hexa­aqua­bis­(μ_4_-pyrimidine-4,6-dicarboxyl­ato)tetra­lithium]

**DOI:** 10.1107/S1600536812038755

**Published:** 2012-09-15

**Authors:** Wojciech Starosta, Janusz Leciejewicz

**Affiliations:** a Institute of Nuclear Chemistry and Technology, ul.Dorodna 16, 03-195 Warszawa, Poland

## Abstract

The asymmetric unit of the title compound, [Li_4_(C_6_H_2_N_2_O_4_)_2_(H_2_O)_6_]_*n*_, comprises two Li^+^ ions bridged by a completely deprotonated pyrimidine-3,6-dicarboxyl­ate ligand and coordinated by two water mol­ecules; the asymmetric units related by an inversion operation create a structural unit which forms part of a two-dimensional polymeric structure parallel to (10-1). One of the Li^+^ ions shows a distorted tetra­hedral arrangement involving two symmetry-related coordinating water mol­ecules and two carboxyl­ate O atoms. The other Li^+^ ion is in distorted trigonal–bipyramidal geometry defined by N and O atoms of the ligands and a water mol­ecule. Water O atoms are proton donors to carboxyl­ate O atoms forming hydrogen bonds.

## Related literature

For the crystal structures of pyrimidine-3,6-dicarb­oxy­lic acid dihydrate and two K^+^ complexes with pyrimidine-3,6-dicarboxyl­ate and aqua ligands, see: Beobide *et al.* (2007 ▶). For the structures of Li^+^ complexes with a pyrimidine-2-carboxyl­ato ligand, see: Starosta & Leciejewicz (2011 ▶) and with a pyrimidine-4-carboxyl­ate ligand, see: Starosta & Leciejewicz (2012 ▶).
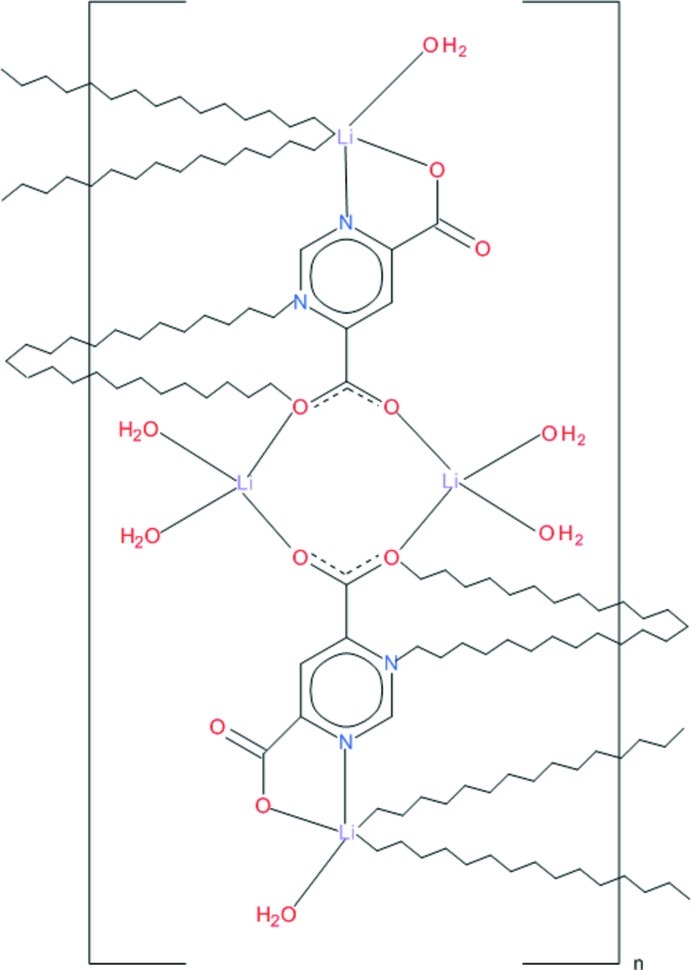


## Experimental

### 

#### Crystal data

[Li_4_(C_6_H_2_N_2_O_4_)_2_(H_2_O)_6_]
*M* *_r_* = 234.02
Monoclinic, 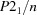

*a* = 6.7014 (13) Å
*b* = 11.755 (2) Å
*c* = 12.251 (3) Å
β = 98.38 (3)°
*V* = 954.8 (3) Å^3^
*Z* = 4
Mo *K*α radiation
μ = 0.15 mm^−1^
*T* = 293 K
0.48 × 0.20 × 0.13 mm

#### Data collection

Kuma KM-4 four-cricle diffractometer
Absorption correction: analytical (*CrysAlis RED*; Oxford Diffraction, 2008 ▶) *T*_min_ = 0.960, *T*_max_ = 0.988
3009 measured reflections
2792 independent reflections
2094 reflections with *I* > 2σ(*I*)
*R*_int_ = 0.023
3 standard reflections every 200 reflections intensity decay: 0.4%

#### Refinement

*R*[*F* ^2^ > 2σ(*F* ^2^)] = 0.037
*wR*(*F* ^2^) = 0.126
*S* = 0.95
2792 reflections
178 parameters
H atoms treated by a mixture of independent and constrained refinement
Δρ_max_ = 0.47 e Å^−3^
Δρ_min_ = −0.41 e Å^−3^

### 

Data collection: *KM-4 Software* (Kuma, 1996 ▶); cell refinement: *KM-4 Software*; data reduction: *DATAPROC* (Kuma, 2001 ▶); program(s) used to solve structure: *SHELXS97* (Sheldrick, 2008 ▶); program(s) used to refine structure: *SHELXL97* (Sheldrick, 2008 ▶); molecular graphics: *SHELXTL* (Sheldrick, 2008 ▶); software used to prepare material for publication: *SHELXTL*.

## Supplementary Material

Crystal structure: contains datablock(s) I, global. DOI: 10.1107/S1600536812038755/kp2437sup1.cif

Structure factors: contains datablock(s) I. DOI: 10.1107/S1600536812038755/kp2437Isup2.hkl

Additional supplementary materials:  crystallographic information; 3D view; checkCIF report

## Figures and Tables

**Table 1:** Selected bond lengths (Å)

Li1—O5	2.081 (3)
Li1—O3^i^	2.100 (2)
Li1—N3^i^	2.153 (2)
Li1—O1	2.030 (2)
Li1—N1	2.156 (2)
Li2—O4^ii^	1.967 (2)
Li2—O7	1.898 (3)
Li2—O6	1.990 (3)
Li2—O3	1.949 (2)

Symmetry codes: (i) 
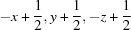
; (ii) 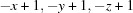.

**Table 2:** Hydrogen-bond geometry (Å, °)

*D*—H⋯*A*	*D*—H	H⋯*A*	*D*⋯*A*	*D*—H⋯*A*
O7—H71⋯O6^iii^	0.85 (3)	2.19 (3)	3.0409 (18)	175 (2)
O7—H72⋯O1^iv^	0.96 (3)	1.75 (3)	2.6920 (15)	168 (2)
O6—H62⋯O2^v^	0.91 (3)	1.85 (3)	2.7518 (15)	172 (2)
O6—H61⋯O5^vi^	0.88 (3)	1.98 (3)	2.7889 (16)	151 (2)
O5—H51⋯O2^vii^	0.87 (3)	1.89 (3)	2.7594 (14)	172 (3)
O5—H52⋯O4^viii^	0.85 (3)	2.02 (3)	2.8670 (14)	178 (2)

Symmetry codes: (iii) 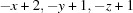; (iv) 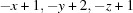; (v) 
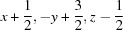
; (vi) 
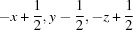
; (vii) 
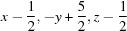
; (viii) 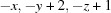.

**Fig. 1.**
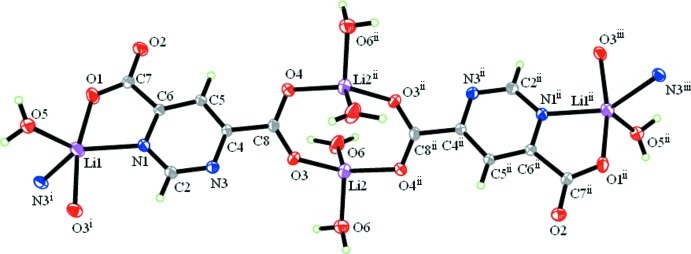
A structural unit of the title compound with atom labelling scheme and 50% probability displacement ellipsoids. Symmetry code: i -x + 1/2, y + 1/2, -z + 1/2; ii -x + 1, -y + 1,-z + 1; iiix + 1/2, -y + 1/2, z + 1/2.

**Fig. 2.**
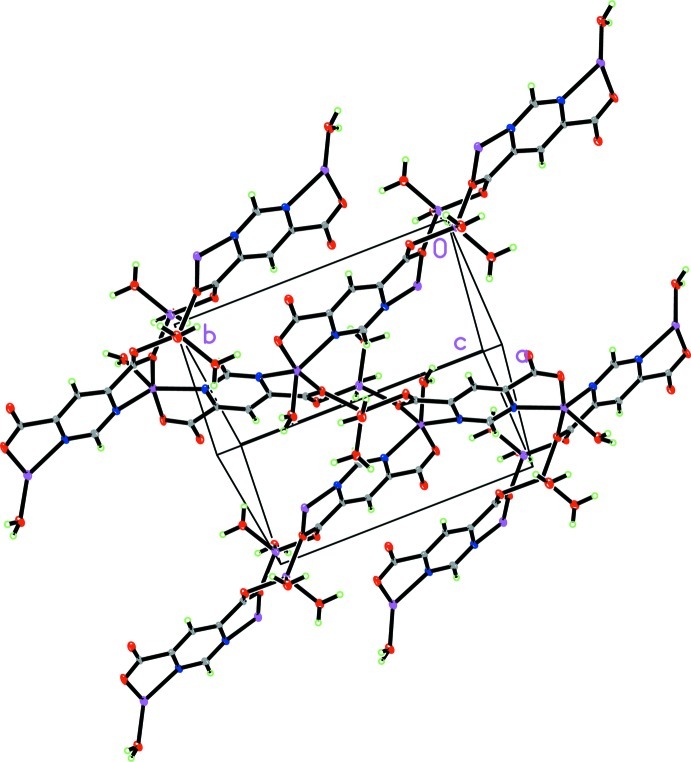
The orientation of a fragment of a single molecular layer in the unit cell of the title structure.

**Fig. 3.**
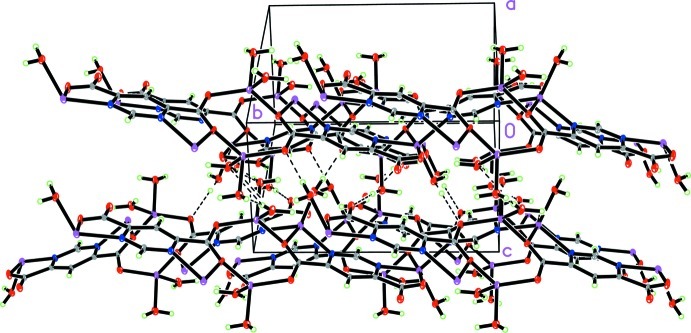
The arrangement of molecular layers in the structure of a LiI complex with pyrimidine-4-carboxylate and aqua molecules.

## supplementary crystallographic information

### Comment

The asymmetric unit of the title compound contains two symmetry independent Li^I^ ions, one with distorted trigonal bipyramidal, the other with distorted tetrahedral coordination geometry, a deprotonated ligand molecule acting in µ_4_ bridging mode and two independent water molecules coordinated to metal ions. The structural unit is built of asymmetric units related by an inversion centre. The ligand N1,O1 bonding group chelates the Li1 ion leaving the carboxylato O2 atom coordination inactive while its carboxylato O3 and O4 atoms bridge, related by an inversion centre, Li2 and Li2^ii^ ions. The latter are also coordinated by O4^ii^ and O3^ii^ atoms donated by the adjacent one related by the same inversion center ligand forming a dimeric bonding loop which constitutes a core of a centrosymmetric structural unit composed of the Li1 ion, the ligand, the dimeric loop, the ligand^ii^ and the Li1^ii^ ion (Fig. 1). Symmetry code; ^i^ -*x* + 1/2, *y* + 1/2, -*z* + 1/2; ^ii^ -*x* + 1, -*y* + 1,-*z* + 1; ^iii^*x* + 1/2, -*y* + 1/2, *z* + 1/2; ^iv^ -*x* + 1/2, *y* - 1/2, -*z* + 1/2; ^v^*x* - 1/2, -*y* + 1/2, *z* - 1/2. The plane of the loop [r.m.s. 0.2647 (5) Å] makes a dihedral angle of 24.0 (2)° with the ligand ring plane. This unit, terminated on both sides by Li1 and Li1^ii^ ions linked to adjacent units *via* N3^i^,O3^i^ and N3^iii^,O3^iii^ bonding groups and *via* N3,O3 and N3^ii^,O3^ii^ bonding groups to Li1^v^ and Li^iv^ions in adjacent units, generate a two-dimensional layer with Li1 ions as its nodes (Fig. 2). The arrangement of the layers in the unit cell is shown in Fig. 3. The coordination polyhedron of the Li1 ion, composed of the bridging N1,O1 and N3^i)^,O3^(i)^ bonding groups and the aqua O5 atom is a distorted trigonal bipyramid. Its equatorial plane is formed by N1, N3^i^ and O5 atoms. The Li1 ion is 0.0908 (2) Å out of this plane, O1 and O3^i^ atoms are at apical positions. The Li2 ion chelated by carboxylato O3,O4^ii^ atoms and aqua O6 with inversion related atoms shows a distorted tetrahedral coordination geometry. The Li—O and Li—N bond lengths (Table 1) fit well to those observed in the structures of Li complexes with other pyrimidine carboxylate ligands (Starosta & Leciejewicz, 2011, 2012). The pyrimidine ring is planar with r.m.s. of 0.0121 (2) A°, C7/O1/O2 and C8/O3/O4 carboxylate groups make with it dihedral angles of 4.0 (1)° and 24.0 (2)°, respectively. Bond distances and bond angles are close to those observed in the structure of the parent acid and its two potassium complexes (Beobide *et al.*, 2007). A network of hydrogen bonds (Table 2), in which coordinated water molecules act as donors and carboxylato O atoms are acceptors maintains the stability of the structure.

### Experimental

1 mmol of pyrimidine-3,6-dicarboxylic acid dihydrate and 2 mmol s of lithium hydroxide were dissolved in 50 mL of hot, doubly distilled water and boiled under reflux with stirring for six hours. Left to crystallize at room temperature, colourless single-crystal blocks deposited after a week. They were washed with cold methanol and dried in the air.

### Refinement

Hydrogen atoms attached to water molecules were located in a difference map and refined isotropically, while two H atoms attached to pyrimidine C atoms were located at a calculated positions and treated as riding on the parent atoms with C—H=0.93 Å and *U*_iso_(H)=1.2*U*_eq_(C).

### Crystal data

**Table:**

[Li_4_(C_6_H_2_N_2_O_4_)_2_(H_2_O)_6_]	*F*(000) = 480
*M**_r_* = 234.02	*D*_x_ = 1.628 Mg m^−^^3^
Monoclinic, *P*2_1_/*n*	Mo *K*α radiation, λ = 0.71073 Å
Hall symbol: -P 2yn	Cell parameters from 25 reflections
*a* = 6.7014 (13) Å	θ = 6–15°
*b* = 11.755 (2) Å	µ = 0.15 mm^−^^1^
*c* = 12.251 (3) Å	*T* = 293 K
β = 98.38 (3)°	Blocks, colourless
*V* = 954.8 (3) Å^3^	0.48 × 0.20 × 0.13 mm
*Z* = 4	

### Data collection

**Table:**

Kuma KM-4 four-cricle diffractometer	2094 reflections with *I* > 2σ(*I*)
Radiation source: fine-focus sealed tube	*R*_int_ = 0.023
Graphite monochromator	θ_max_ = 30.1°, θ_min_ = 2.4°
profile data from ω/2θ scans	*h* = −9→0
Absorption correction: analytical (*CrysAlis RED*; Oxford Diffraction, 2008)	*k* = 0→16
*T*_min_ = 0.960, *T*_max_ = 0.988	*l* = −17→17
3009 measured reflections	3 standard reflections every 200 reflections
2792 independent reflections	intensity decay: 0.4%

### Refinement

**Table:**

Refinement on *F*^2^	Primary atom site location: structure-invariant direct methods
Least-squares matrix: full	Secondary atom site location: difference Fourier map
*R*[*F*^2^ > 2σ(*F*^2^)] = 0.037	Hydrogen site location: inferred from neighbouring sites
*wR*(*F*^2^) = 0.126	H atoms treated by a mixture of independent and constrained refinement
*S* = 0.95	*w* = 1/[σ^2^(*F*_o_^2^) + (0.0949*P*)^2^ + 0.1908*P*] where *P* = (*F*_o_^2^ + 2*F*_c_^2^)/3
2792 reflections	(Δ/σ)_max_ = 0.001
178 parameters	Δρ_max_ = 0.47 e Å^−^^3^
0 restraints	Δρ_min_ = −0.41 e Å^−^^3^

### Special details

**Table:**

Geometry. All e.s.d.'s (except the e.s.d. in the dihedral angle between two l.s. planes) are estimated using the full covariance matrix. The cell e.s.d.'s are taken into account individually in the estimation of e.s.d.'s in distances, angles and torsion angles; correlations between e.s.d.'s in cell parameters are only used when they are defined by crystal symmetry. An approximate (isotropic) treatment of cell e.s.d.'s is used for estimating e.s.d.'s involving l.s. planes.
Refinement. Refinement of *F*^2^ against ALL reflections. The weighted *R*-factor *wR* and goodness of fit *S* are based on *F*^2^, conventional *R*-factors *R* are based on *F*, with *F* set to zero for negative *F*^2^. The threshold expression of *F*^2^ > σ(*F*^2^) is used only for calculating *R*-factors(gt) *etc*. and is not relevant to the choice of reflections for refinement. *R*-factors based on *F*^2^ are statistically about twice as large as those based on *F*, and *R*- factors based on ALL data will be even larger.

### Fractional atomic coordinates and isotropic or equivalent isotropic displacement parameters (Å^2^)

**Table:**

	*x*	*y*	*z*	*U*_iso_*/*U*_eq_	
O2	0.24613 (17)	1.10050 (8)	0.59876 (7)	0.0279 (2)	
O3	0.44978 (14)	0.61780 (7)	0.39479 (7)	0.0225 (2)	
O4	0.34896 (15)	0.65464 (8)	0.55698 (7)	0.0244 (2)	
N3	0.26576 (17)	0.80458 (9)	0.30736 (8)	0.0208 (2)	
O1	0.16917 (19)	1.18808 (8)	0.43666 (8)	0.0321 (2)	
C8	0.37372 (17)	0.67999 (9)	0.46084 (9)	0.0174 (2)	
N1	0.18480 (16)	1.00000 (8)	0.32426 (8)	0.0205 (2)	
C7	0.21347 (17)	1.10298 (9)	0.49612 (9)	0.0181 (2)	
O6	0.82841 (18)	0.51649 (10)	0.29471 (8)	0.0315 (2)	
C4	0.30347 (16)	0.79650 (9)	0.41748 (9)	0.0162 (2)	
C6	0.22823 (16)	0.99180 (9)	0.43410 (9)	0.0157 (2)	
O7	0.85977 (18)	0.58692 (9)	0.52784 (9)	0.0345 (3)	
C5	0.28409 (18)	0.88905 (9)	0.48558 (9)	0.0172 (2)	
H5	0.3074	0.8825	0.5620	0.021*	
C2	0.2031 (2)	0.90580 (10)	0.26635 (9)	0.0235 (3)	
H2	0.1691	0.9109	0.1902	0.028*	
Li1	0.0920 (4)	1.16918 (19)	0.27116 (17)	0.0257 (5)	
Li2	0.6732 (4)	0.51046 (19)	0.4213 (2)	0.0259 (4)	
H71	0.951 (5)	0.556 (2)	0.574 (2)	0.070 (8)*	
H72	0.848 (4)	0.668 (2)	0.530 (2)	0.056 (7)*	
H62	0.789 (4)	0.481 (2)	0.229 (2)	0.058 (7)*	
H61	0.832 (4)	0.589 (2)	0.279 (2)	0.055 (7)*	
O5	−0.19709 (17)	1.23656 (9)	0.26248 (8)	0.0291 (2)	
H51	−0.223 (4)	1.284 (2)	0.207 (2)	0.066 (7)*	
H52	−0.239 (4)	1.268 (2)	0.317 (2)	0.055 (6)*	

### Atomic displacement parameters (Å^2^)

**Table:**

	*U*^11^	*U*^22^	*U*^33^	*U*^12^	*U*^13^	*U*^23^
O2	0.0469 (6)	0.0212 (4)	0.0151 (4)	0.0015 (4)	0.0028 (4)	−0.0020 (3)
O3	0.0321 (5)	0.0169 (4)	0.0189 (4)	0.0065 (3)	0.0052 (3)	−0.0018 (3)
O4	0.0391 (5)	0.0177 (4)	0.0176 (4)	0.0048 (3)	0.0080 (3)	0.0036 (3)
N3	0.0309 (5)	0.0173 (4)	0.0140 (4)	0.0046 (4)	0.0021 (4)	−0.0008 (3)
O1	0.0598 (7)	0.0139 (4)	0.0214 (4)	0.0058 (4)	0.0022 (4)	0.0013 (3)
C8	0.0217 (5)	0.0126 (4)	0.0174 (5)	0.0013 (4)	0.0013 (4)	0.0006 (4)
N1	0.0310 (5)	0.0162 (4)	0.0143 (4)	0.0049 (4)	0.0029 (4)	0.0017 (3)
C7	0.0229 (5)	0.0142 (5)	0.0175 (5)	0.0005 (4)	0.0037 (4)	−0.0018 (4)
O6	0.0486 (6)	0.0248 (5)	0.0221 (5)	0.0005 (4)	0.0090 (4)	−0.0011 (4)
C4	0.0205 (5)	0.0134 (4)	0.0149 (5)	0.0018 (4)	0.0031 (4)	0.0011 (3)
C6	0.0200 (5)	0.0134 (5)	0.0142 (5)	0.0007 (4)	0.0040 (4)	−0.0001 (4)
O7	0.0406 (6)	0.0243 (5)	0.0359 (6)	0.0057 (4)	−0.0034 (4)	−0.0093 (4)
C5	0.0249 (5)	0.0147 (5)	0.0123 (4)	0.0024 (4)	0.0035 (4)	0.0007 (4)
C2	0.0374 (7)	0.0195 (5)	0.0128 (5)	0.0065 (5)	0.0013 (4)	0.0002 (4)
Li1	0.0381 (12)	0.0196 (10)	0.0195 (10)	−0.0007 (8)	0.0042 (9)	0.0028 (8)
Li2	0.0334 (11)	0.0186 (10)	0.0261 (10)	0.0034 (8)	0.0053 (8)	0.0000 (8)
O5	0.0445 (6)	0.0217 (4)	0.0228 (4)	0.0065 (4)	0.0102 (4)	0.0028 (4)

### Geometric parameters (Å, º)

**Table:**

O2—C7	1.2449 (14)	C8—Li2^ii^	2.707 (3)
O3—C8	1.2527 (14)	N1—C2	1.3305 (15)
Li1—O5	2.081 (3)	N1—C6	1.3382 (14)
Li1—O3^i^	2.100 (2)	C7—C6	1.5222 (15)
Li1—N3^i^	2.153 (2)	O6—H62	0.91 (3)
Li1—O1	2.030 (2)	O6—H61	0.88 (3)
Li1—N1	2.156 (2)	C4—C5	1.3886 (15)
Li2—O4^ii^	1.967 (2)	C6—C5	1.3885 (15)
Li2—O7	1.898 (3)	O7—H71	0.85 (3)
Li2—O6	1.990 (3)	O7—H72	0.96 (3)
Li2—O3	1.949 (2)	C5—H5	0.9300
O3—Li1^iii^	2.100 (2)	C2—H2	0.9300
O4—C8	1.2492 (14)	Li1—Li2^i^	3.313 (3)
O4—Li2^ii^	1.968 (2)	Li2—C8^ii^	2.707 (3)
N3—C2	1.3352 (15)	Li2—Li2^ii^	3.237 (5)
N3—C4	1.3393 (14)	Li2—Li1^iii^	3.313 (3)
N3—Li1^iii^	2.153 (2)	O5—H51	0.87 (3)
O1—C7	1.2476 (14)	O5—H52	0.85 (3)
C8—C4	1.5187 (15)		
			
C8—O3—Li2	130.20 (11)	O1—Li1—O3^i^	167.65 (14)
C8—O3—Li1^iii^	116.62 (10)	O5—Li1—O3^i^	93.91 (10)
Li2—O3—Li1^iii^	109.76 (11)	O1—Li1—N1	77.25 (8)
C8—O4—Li2^ii^	112.72 (10)	O5—Li1—N1	126.29 (12)
C2—N3—C4	116.43 (10)	O3^i^—Li1—N1	91.07 (10)
C2—N3—Li1^iii^	128.76 (10)	O1—Li1—N3^i^	107.48 (11)
C4—N3—Li1^iii^	111.52 (9)	O5—Li1—N3^i^	99.51 (10)
C7—O1—Li1	119.98 (10)	O3^i^—Li1—N3^i^	77.60 (8)
O4—C8—O3	126.32 (11)	N1—Li1—N3^i^	133.62 (12)
O4—C8—C4	117.91 (10)	O1—Li1—Li2^i^	143.55 (11)
O3—C8—C4	115.77 (10)	O5—Li1—Li2^i^	77.24 (9)
O4—C8—Li2^ii^	42.09 (7)	O3^i^—Li1—Li2^i^	33.61 (6)
O3—C8—Li2^ii^	87.24 (8)	N1—Li1—Li2^i^	78.26 (8)
C4—C8—Li2^ii^	151.42 (9)	N3^i^—Li1—Li2^i^	108.96 (9)
C2—N1—C6	116.92 (10)	O7—Li2—O3	102.77 (12)
C2—N1—Li1	130.69 (10)	O7—Li2—O4^ii^	115.41 (13)
C6—N1—Li1	112.39 (9)	O3—Li2—O4^ii^	126.15 (14)
O1—C7—O2	126.90 (11)	O7—Li2—O6	98.77 (12)
O1—C7—C6	115.10 (10)	O3—Li2—O6	108.87 (12)
O2—C7—C6	118.00 (10)	O4^ii^—Li2—O6	101.48 (11)
Li2—O6—H62	124.0 (15)	O7—Li2—C8^ii^	98.08 (10)
Li2—O6—H61	104.1 (16)	O3—Li2—C8^ii^	118.56 (11)
H62—O6—H61	105 (2)	O4^ii^—Li2—C8^ii^	25.19 (5)
N3—C4—C5	121.96 (10)	O6—Li2—C8^ii^	124.03 (11)
N3—C4—C8	114.82 (9)	O7—Li2—Li2^ii^	94.80 (12)
C5—C4—C8	123.20 (10)	O3—Li2—Li2^ii^	63.13 (9)
N1—C6—C5	121.62 (10)	O4^ii^—Li2—Li2^ii^	76.67 (10)
N1—C6—C7	114.81 (9)	O6—Li2—Li2^ii^	165.63 (16)
C5—C6—C7	123.56 (10)	C8^ii^—Li2—Li2^ii^	58.00 (7)
Li2—O7—H71	126.2 (19)	O7—Li2—Li1^iii^	117.02 (11)
Li2—O7—H72	116.1 (14)	O3—Li2—Li1^iii^	36.62 (6)
H71—O7—H72	118 (2)	O4^ii^—Li2—Li1^iii^	127.50 (11)
C4—C5—C6	116.82 (10)	O6—Li2—Li1^iii^	73.31 (8)
C4—C5—H5	121.6	C8^ii^—Li2—Li1^iii^	138.88 (10)
C6—C5—H5	121.6	Li2^ii^—Li2—Li1^iii^	96.31 (11)
N1—C2—N3	126.12 (10)	Li1—O5—H51	111.2 (18)
N1—C2—H2	116.9	Li1—O5—H52	122.7 (17)
N3—C2—H2	116.9	H51—O5—H52	106 (2)
O1—Li1—O5	96.27 (11)		

Symmetry codes: (i) −*x*+1/2, *y*+1/2, −*z*+1/2; (ii) −*x*+1, −*y*+1, −*z*+1; (iii) −*x*+1/2, *y*−1/2, −*z*+1/2.

### Hydrogen-bond geometry (Å, º)

**Table:**

*D*—H···*A*	*D*—H	H···*A*	*D*···*A*	*D*—H···*A*
O7—H71···O6^iv^	0.85 (3)	2.19 (3)	3.0409 (18)	175 (2)
O7—H72···O1^v^	0.96 (3)	1.75 (3)	2.6920 (15)	168 (2)
O6—H62···O2^vi^	0.91 (3)	1.85 (3)	2.7518 (15)	172 (2)
O6—H61···O5^iii^	0.88 (3)	1.98 (3)	2.7889 (16)	151 (2)
O5—H51···O2^vii^	0.87 (3)	1.89 (3)	2.7594 (14)	172 (3)
O5—H52···O4^viii^	0.85 (3)	2.02 (3)	2.8670 (14)	178 (2)

Symmetry codes: (iii) −*x*+1/2, *y*−1/2, −*z*+1/2; (iv) −*x*+2, −*y*+1, −*z*+1; (v) −*x*+1, −*y*+2, −*z*+1; (vi) *x*+1/2, −*y*+3/2, *z*−1/2; (vii) *x*−1/2, −*y*+5/2, *z*−1/2; (viii) −*x*, −*y*+2, −*z*+1.
